# Annulus fibrosus functional extrafibrillar and fibrous mechanical behaviour: experimental and computational characterisation

**DOI:** 10.1098/rsos.170807

**Published:** 2017-08-23

**Authors:** Marlène Mengoni, Oluwasegun Kayode, Sebastien N. F. Sikora, Fernando Y. Zapata-Cornelio, Diane E. Gregory, Ruth K. Wilcox

**Affiliations:** 1 Institute of Medical and Biological Engineering, School of Mechanical Engineering, University of Leeds, Leeds, UK; 2 Department of Kinesiology and Physical Education, Wilfrid Laurier University, Waterloo, Ontario, Canada

**Keywords:** intervertebral disc, annulus fibrosus, pre-strain, reverse engineering, direct-controlled calibration

## Abstract

The development of current surgical treatments for intervertebral disc damage could benefit from virtual environment accounting for population variations. For such models to be reliable, a relevant description of the mechanical properties of the different tissues and their role in the functional mechanics of the disc is of major importance. The aims of this work were first to assess the physiological hoop strain in the annulus fibrosus in fresh conditions (*n* = 5) in order to extract a functional behaviour of the extrafibrillar matrix; then to reverse-engineer the annulus fibrosus fibrillar behaviour (*n* = 6). This was achieved by performing both direct and global controlled calibration of material parameters, accounting for the whole process of experimental design and *in silico* model methodology. Direct-controlled models are specimen-specific models representing controlled experimental conditions that can be replicated and directly comparing measurements. Validation was performed on another six specimens and a sensitivity study was performed. Hoop strains were measured as 17 ± 3% after 10 min relaxation and 21 ± 4% after 20–25 min relaxation, with no significant difference between the two measurements. The extrafibrillar matrix functional moduli were measured as 1.5 ± 0.7 MPa. Fibre-related material parameters showed large variability, with a variance above 0.28. Direct-controlled calibration and validation provides confidence that the model development methodology can capture the measurable variation within the population of tested specimens.

## 1. Introduction

Degeneration or trauma of intervertebral discs accounts for almost half of causal diagnosis of low back pain [1]. However, current surgical treatments are highly invasive and have low long-term success rates, with limited methods available for the preclinical evaluation of novel therapies. A virtual environment has the potential to be used as a tool for targeting patients or testing novel treatments for the disc accounting for population variations. It can provide systematic methods to analyse the outcome of a therapy and its potential for success, to inform experimental testing and target key factors needing control, to test scenario accounting for patient-specific or disease-specific changes in anatomy and tissue behaviour. For such models to be reliable in a clinical or product development environment, a relevant description of the mechanical properties of the different tissues and their role in the functional mechanics of the disc is of major importance.

Material behaviour of the annulus has recently been modelled with a linear [2–5] or nonlinear [6–8] extrafibrillar matrix embedded with linear [6,8] or nonlinear [2–5,7] oriented fibres acting in tension only. Material parameters are often derived from experimental data of excised tissue, e.g. the behaviour of the fibres [4,9–12] or the extrafibrillar matrix (EFM) [11–13] in the annulus fibrosus have been derived from mechanical tests performed on specimens of single lamellae that have been prepared so that load is either oriented in the direction of the fibres or perpendicular to them. While these approaches have enabled the individual component properties to be derived, they necessarily have the disadvantage that the collected data do not account for the *in situ* state of the tissue, such as the pre-strain, inter-lamellar connectivity, or level of hydration. Moreover, increasingly complex biomechanical models [2,4] have been recently developed, some directly calibrated against experimental data [4], with the drawback that a low number of measurements is sometimes used to calibrate a relatively high number of material parameters, therefore not always convincingly with unique solution. The role of residual stress or pre-strain in soft tissues has been highlighted early in the literature [14] but only limited work has been done on evaluating pre-strain in the annulus [15,16], and, to the authors' knowledge, none on fresh tissue.

The aims of this work were first to assess the physiological hoop strain in the annulus fibrosus with respect to its excised stress–strain extrafibrillar matrix behaviour, in order to extract a functional or physiologically relevant range and behaviour; then to reverse-engineer the annulus fibrosus fibrillar behaviour, using image-specific finite-element models and direct-controlled calibration [17]. Here, the terms *direct* calibration and validation are used to define processes where specimen-specific models derived from corresponding experimental (*in vitro* or *in vivo*) data are employed and the same measures are compared in the models and experiments. The terms *controlled* calibration and validation are used to define the way in which the experimental data is acquired, such that it enables an exact replication of loads and boundary conditions in the model; this is typically data acquired *in vitro*.

## 2. Material and methods

Bovine caudal tissue from abattoir animals between 2 and 3 years old were used for all parts of this study. Bovine caudal tissue was used because it is available as young healthy tissue, and the caudal functional unit does not include facet joints, making its testing relevant to the behaviour of the intervertebral disc and not affected by the balance between three different joints.

### 2.1. Functional extrafibrillar matrix behaviour

The functional extrafibrillar matrix behaviour was defined as the mechanical behaviour of the matrix components of the annulus fibrosus, measured in the range of strains that are physiological and accounting for hoop strain of the tissue.

#### 2.1.1. Physiological hoop strain assessment

**Figure 1.**
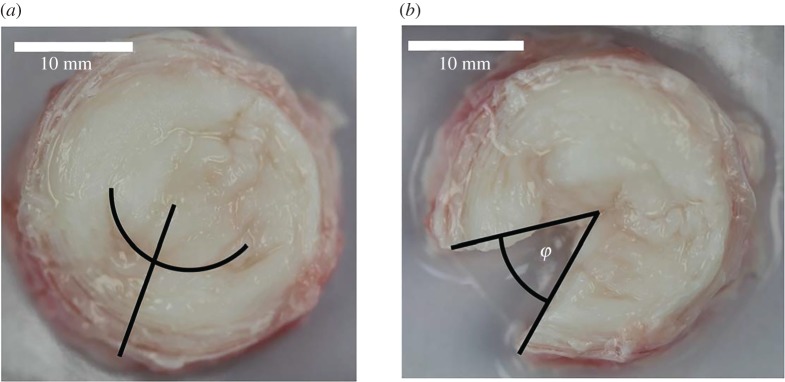
(*a*) Intact intervertebral disc with location and extent of the circumferential and radial cuts; (*b*) intervertebral disc after 10 min opening relaxation.

Intervertebral discs (*n* = 5) were harvested from levels c1-2 to c5-6 of two tails obtained freshly from the local abattoir. The tails were prepared on the day of collection to isolate the c1-c6 section and soft tissues were removed, avoiding damage to the fat capsule surrounding the intervertebral discs. Tail sections were wrapped into PBS-soaked tissue, sealed in plastic bags, and kept in a fridge overnight. On the following day, the sections were dissected to isolate the intervertebral discs from the bone, separating the intervertebral discs from the vertebrae with a scalpel blade, closely following endplate boundaries (figure 1*a*).

Annulus tissue was freed from the central nucleus pulposus with a circumferential cut spanning an arc of about 90° angle in the inner annulus (figure 1*a*), at a circumferential location where the annulus was the thickest radially and clearer tissue boundaries could be identified. A radial cut through the annulus was performed centrally to that first incision, and the disc was left free to open. High resolution photographs with a scale were taken of the intact discs and at two time points after the radial incision, after 10 min and between 20 and 25 min. Following preliminary tests, it was found that hydration and friction were important factors in the opening of the discs. The intervertebral discs were therefore sprayed with PBS throughout the procedure to keep them hydrated and to maintain a low friction interface with the surface they rested on.

Intact annulus and nucleus diameters and angular opening of the annulus, *φ* (figure 1*b*), were measured in Fiji/ImageJ 1.5.1e [18]. All measurements were performed six times, to account for small variations in the manual identification of key characteristics (definition of the centre of the disc, angle at which the diameters were measured, and location of the edge of the cuts), and average measurements were recorded. Nucleus-to-annulus diameter ratio were extracted and hoop strain, i.e. the strain needed for the opened annulus to be closed, was derived as the outer annulus hoop strain $\varepsilon_{h}=\varphi /(2\pi -\varphi )$, assuming circularity of the disc and disc diameter maintained with tissue relaxation. Circularity deviation of the intervertebral discs was measured as the standard deviation of the ratio of the six measured diameters to their mean value. This definition of circularity deviation gives a value of 0 for a perfect circle, 0.24 for an ellipse with axis ratio of 1 : 2, and 0.592 as the aspect ratio tends to infinity (assuming measurements equally distributed). This was computed for the initial geometry of the disc and for its state at 10 min relaxation, for which two of the diameter measurements corresponded to tangents to the edge of the cut.

#### 2.1.2. Extrafibrillar matrix mechanical behaviour

Extrafibrillar matrix tensile data were obtained from single annulus lamellae tested in a previous study [19]: single lamellae were dissected from the annulus and tested in tension in the direction perpendicular to the fibres (*n* = 16); the tissue was preconditioned for three cycles then loaded until failure or 300% strains. The measured loads and displacements were converted into engineering strain and engineering stress using the length of the tissue at the end of preconditioning and the initial cross-sectional area of the tissue.

A bespoke script in MATLAB (R2014b, The Mathworks Inc., Natick, MA, USA) was used to define the strain range between the hoop strain $\varepsilon_{h}$ measured in §2.1.1 and the failure strain. A linear fit of the stress–strain data in that region was used to define the extrafibrillar matrix functional modulus.

### 2.2. Reverse-engineering of fibrillar mechanical behaviour

Twelve specimen-specific finite elements models of bovine osteodiscs (disc surrounded by two half vertebrae) were produced from micro-CT images, and material model parameters for the annulus fibrosus fibres were directly calibrated against experimental data obtained in controlled conditions on the same specimens. Results from the first part of the study were used to describe the extrafibrillar matrix behaviour of the annulus, reducing the number of parameters to calibrate to those just describing the fibrillar component of the annulus.

#### 2.2.1. Osteodiscs specimen preparation, mechanical testing and imaging

In a parallel study [20], osteodiscs (*n* = 12) were harvested from levels c1-2 to c4-5 of eight tails obtained freshly from the local abattoir. The tails were dissected and prepared to isolate osteodiscs with a consistent bone thickness of 15 mm either side of the intervertebral disc and potted into PMMA endcaps fitting compression test fixtures. The specimens were imaged using a micro-CT scanner (micro-CT 100, Scanco Medical AG, Switzerland) at an isotropic voxel size of 74 microns.

The specimens were tested in quasi-static axial compression with a materials testing machine (Electropuls E10000, Instron, USA). Transverse translations were kept free by fixing the specimen to the cross-head via two linear bearings. Axial compression was obtained at a rate of 1 mm min^−1^ up to a load of 2100 N. Displacements were measured with respect to the displacement at a load of 10 N, corresponding to the weight of the compression plate.

#### 2.2.2. Finite-element modelling with reverse-engineering

**Figure 2.**
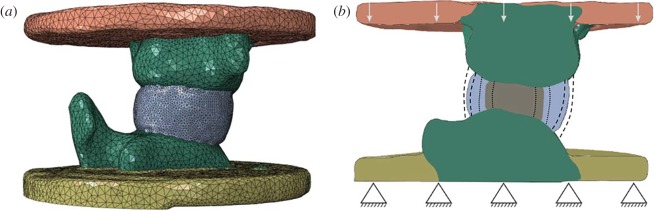
(*a*) Image-based finite-element mesh of an osteodisc specimen; (*b*) boundary conditions and disc geometry variation for sensitivity studies.

Finite-element models of caudal bovine osteodiscs were built from the micro-CT images (figure 2*a*). The micro-CT images were scaled to 0–255 greyscale and specimen-specific geometries were derived using image processing tools in Simpleware ScanIP 7.0 (Synopsys, Mountain View, USA) with images down-sampled to an isotropic 0.5 mm resolution. As soft tissues cannot be distinguished in the micro-CT images, a systematic protocol was developed to create the annulus and nucleus regions of the disc based on the diameter ratio measured in the first part of this study and assuming relatively straight sides from one endplate to the other, which is a good approximation for bovine caudal discs [8]. Finite-element models were produced using the segmented geometry and underlying greyscale, with a homogeneous linear tetrahedral mesh with a size based on previous studies [5].

Boundary conditions replicating the experimental tests were applied. The outer surface of the distal endcap was clamped, and an axial translation, of the same magnitude as the corresponding *in vitro* displacement, was applied to the outer surface of the proximal endcap whereas all other translations were kept free and all rotations were restricted (figure 2*b*).

Material behaviour for the bone was based on the greyscale of the image, using element-by-element Young's modulus values linearly dependent on the underlying average greyscale [21], with a conversion factor calibrated and validated for bovine caudal vertebrae in a parallel study. This resulted in elastic modulus values in the range of 10–910 MPa, with a volume average of 409 MPa. The bone Poisson's ratio was assumed to be 0.3. PMMA cement was modelled as linear elastic with a Young's modulus of 1.5 GPa and a Poisson's ratio of 0.3. The nucleus was modelled as an incompressible Mooney–Rivlin material [6], where the incompressibility in static compression comes mainly from the nucleus's high water content. Hybrid linear element integration was used for the nucleus pulposus.

The annulus non-linearity was captured with a GOH exponential model [22] assuming two oblique/counter-oblique fibre orientations at 20° to the transverse plane [23], and perfect directionality (*κ* = 0). The extrafibrillar matrix functional modulus derived in the first part of the study was used (leading to *C*_10_ = 0.25 MPa), and compressibility was that of water (*D* = 9.09 × 10^−4^ MPa^−1^). The 12 specimens were separated in two groups of 6, one for calibration of the two material parameters describing the exponential fibre behaviour (*k*_1_, in MPa, and *k*_2_), and one for validation. For the calibration group, two methods were used to derive the fibre behaviour: first, a specimen-specific calibration, using a reverse-engineering method that minimizes, for each specimen, the RMS difference between the *in vitro* and *in silico* loads, deriving a pair of material parameters for every specimen; and second, an average calibration, using the same reverse-engineering method but minimizing the average RMS difference over the six specimens, deriving a single pair of material parameters for the whole set. Reverse-engineering was performed with an L-BFGS-B algorithm [24], using the opti4abq toolbox [25,26]. The RMS difference was used rather than the RMS error in order to reduce the effects of small boundary conditions discrepancy at low displacements. The calibration process was considered successful when either the RMS difference was below 5% of the max applied load (i.e. less than 105 N) or the value of the parameters varied by less than 5% between two iterations. Validation was performed on the other group of specimens using the parameters derived in the average calibration. For comparison purposes, all twelve specimens were also modelled using values derived in another bovine disc study [7].

Four sensitivity studies were performed on all specimens of the calibration group: two on the material parameters for the nucleus, either modifying its compressibility or its equivalent modulus, and two on the geometry of the disc, either modifying its size or the nucleus-to-annulus diameter ratio (figure 2*b*). All other parameters and boundary conditions were kept as described hitherto. Annulus fibres material parameters used for these four studies were those obtained from the literature [7]. For the nucleus compressibility study, the nucleus material model was altered from incompressible to a Poisson's ratio of 0.4999 (a bulk modulus equivalent to that of water) or to a slightly more compressible value of 0.49. For the nucleus modulus study, the two material parameters of the Mooney–Rivlin model were altered to 0.1 and 10 times their baseline values, keeping the nucleus incompressible. For the disc size sensitivity study, the external disc diameter were increased or decreased by 5–15%, keeping an overall appearance of the discs anatomically consistent with the shape of the endplates, and a nucleus-to-annulus diameter ratio constant. For the nucleus-to-annulus ratio sensitivity study, the diameter of the nucleus was varied to be 0.4, 0.5 (baseline) or 0.6 times the disc diameter measured centrally in the cranial-caudal direction, keeping the nucleus centred in the axial plane.

All finite-element analyses were nonlinear quasi-static and run in parallel with ABAQUS 6.14 (Simulia, Dassault Système). All osteodisc specimens showed an experimental or computational force-displacement behaviour with an initial toe region and a final linear zone. A custom-written MATLAB algorithm was used to determine the gradients of a continuous tri-linear fit, defining three stiffness values (initial, transition and linear), and the two transition displacements between the three zones.

### 2.3. Statistical analysis

The hoop strains values measured at 10 and 20–25 min were compared using a two-sided paired Student *t*-test, after the normal nature of the data was assessed with a Shapiro–Wilk test. The same tests were performed for the deviation of circularity between intact state and 10 min relaxation, for the diameter at 10 min relaxation between the average of the two measures along the edges of the cut and the average of the other four measures, and for the mean diameter between intact state and the 10 min relaxation state.

Pearson's correlation coefficients were used to measure the linearity of the stress–strain data of single annulus lamellae tested in the strain range of interest.

The agreement between experimental and computational stiffness values of the tri-linear fit were assessed with concordance correlation coefficients (CCC). A similar comparison was performed between the stiffness values obtained for the four sensitivity studies and the corresponding baseline models.

All statistical analyses were performed with R v. 3.1.2 (R Foundation for Statistical Computing) and statistical significance was set at *p* < 0.05.

## 3. Results

All new raw and processed data produced in this study, including processing scripts in R and MATLAB, are available openly from the University of Leeds data repository [27].

### 3.1. Functional extrafibrillar matrix behaviour

**Figure 3.**
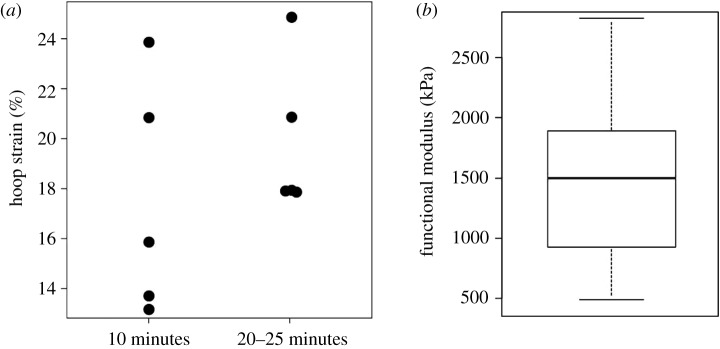
(*a*) Hoop strain measurements at 10 min relaxation and between 20 and 25 min (*n* = 5); (*b*) box plot of the functional modulus values (*n* = 16).

The nucleus-to-annulus diameter ratio was measured (mean ± s.d.) as 0.51 ± 0.05. Deviation from perfect circle was below 0.06 for all five discs, in intact conditions and after 10 min relaxation. No significant differences were observed between the two measures (*p* = 0.14). Further, no significant differences were observed between the two diameters measured at the edge of the cut at 10 min relaxation and the other four measurements (*p* = 0.07), or between average diameters in intact conditions and after 10 min relaxation (*p* = 0.25). Hoop strains were measured as 17 ± 3% after 10 min relaxation and 21 ± 4% after 20–25 min relaxation (figure 3*a*). No significant difference was observed between the two hoop strain measurements (*p* = 0.07).

The extrafibrillar matrix functional moduli were measured as 1.5 ± 0.7 MPa (figure 3*b*). All specimens presented highly linear mechanical behaviour for strains higher than the hoop strain, with Pearson's correlation coefficients between 0.954 and 0.999.

### 3.2. Reverse-engineering of fibrillar mechanical behaviour

The material calibration performed separately on each model led to a convergence of the RMS difference value for four models and of the parameter values for the two remaining models (table 1). The material calibration performed on the set of six specimens converged for the parameter values, not the RMS difference.

**Table 1.** Annulus fibres material coefficients for the exponential part of a GHO model, with RMS difference values and CCC (with 95% confidence intervals), slope and intercept for all stiffness values with respect to experimental data (figure 4*b*).

	*k*_1_ (MPa)	*k*_2_	RMS diff (N)	CCC	slope	intercept (N mm^−1^)
calibration group (*n* = 6)						
specimen-specific calibration				0.855 (0.687–0.936)	0.91	−178
Specimen 1	1.41	1.44	216			
Specimen 2	2.69	2.63	65			
Specimen 3	1.94	1.79	64			
Specimen 4	1.75	1.80	97			
Specimen 5	0.60	1.10	454			
Specimen 6	1.27	1.33	87			
average calibration (set of six specimens)						
	1.43	1.63	320	0.701 (0.373–0.873)	1.55	161
literature [7]						
	2.45	2.1	775	0.607 (0.386–0.762)	1.96	84
validation group (*n* = 6)						
average calibration	1.43	1.63	670	0.570 (0.255–0.776)	1.87	108
literature [7]	2.45	2.1	961	0.473 (0.202–0.673)	2.24	106

**Figure 4.**
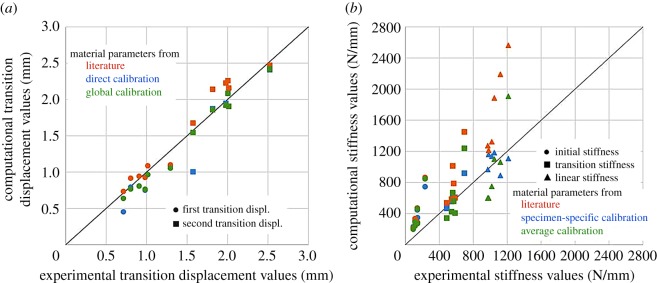
Agreement between computational and experimental values, function of the material coefficients for the annulus fibres: (*a*) transition displacement values; (*b*) stiffness values.

In the calibration group, models with material parameters from the literature resulted in a very poor concordance with the experimental data (table 1 and figure 4) for all stiffness values. Specimen-specific calibration models resulted in a substantial concordance for all stiffness values. Average calibration models resulted in a poor concordance for the initial stiffness value and a moderate concordance for the transition and linear stiffness values.

The RMS difference and the concordance in the validation group improved with respect to models with material parameters from the literature (table 1).

**Figure 5.**
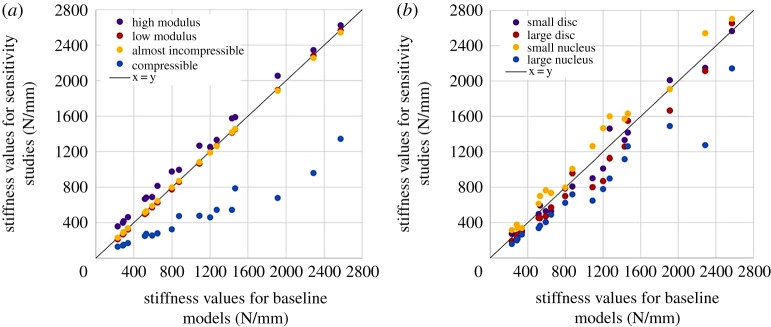
Agreement between computational values for the nucleus sensitivity studies and their baseline computational equivalent: (*a*) effect of nucleus model; (*b*) effect of disc geometry.

**Figure 6.**
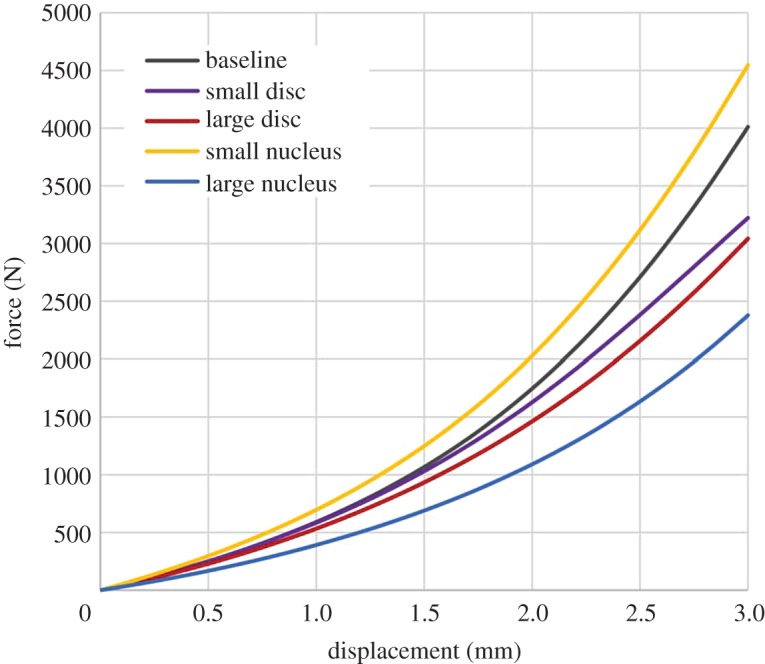
Example of force displacement behaviour for all four geometry sensitivity studies.

Using an almost incompressible nucleus (compressibility of water) did not make any difference to the computational results, whereas increasing the compressibility (Poisson's ratio 0.49) decreased the stiffness values (table 2 and figure 5*a*). Increasing the material parameters of the Mooney–Rivlin model for the nucleus by one order of magnitude increased the values of the apparent stiffness (table 2 and figure 5*a*). Decreasing the same parameters by the same amount showed no difference with their baseline values. Changing the size of the intervertebral disc diameter did not have any significant effect on stiffness values (table 2 and figure 5*b*). Decreasing the nucleus-to-annulus diameter ratio slightly increased the stiffness values while increasing the ratio significantly decreased them (table 2 and figure 5*b*). A summary of the load–displacement data for the geometry sensitivity models of one randomly chosen specimen is given in figure 6.

**Table 2.** Sensitivity for the nucleus material model and geometry of the disc, CCC (with 95% confidence intervals), slope and intercept for all stiffness values with respect to the baseline computational model (figure 5).

	CCC	slope	intercept (N mm^−1^)
nucleus model			
almost incompressible	0.999	0.99	−6
low compressibility	0.471 (0.282–0.624)	0.48	55
lower equivalent modulus	0.999	1.003	−18
higher equivalent modulus	0.982 (0.963–0.991)	0.97	151
disc geometry			
smaller disc	0.989 (0.972–0.996)	1.01	48
larger disc	0.976 (0.941–0.991)	1.01	−99
smaller NP : AF	0.974 (0.943–0.989)	1.06	65
larger NP : AF	0.847 (0.711–0.922)	0.79	−79

## 4. Discussion

In this study, a combined experimental and computational approach was used to derive the properties of the different components of the annulus fibrosis. Physiological hoop strain in the annulus fibrosus was measured in fresh tissue for the first time, and a functional or physiological mechanical behaviour was defined for its extrafibrillar matrix. Specimen-specific finite-element models of osteodiscs were built and the annulus fibrous behaviour was calibrated against experimental data in controlled conditions, showing a substantial agreement of stiffness variation with increasing deformations.

### 4.1. Hoop strain and functional extrafibrillar matrix behaviour

All the specimens were circular, as already reported for bovine discs [28,29], and stayed circular without diameter change with relaxation. In their relaxed state, they showed cuts staying radial and straight with no local change in diameter, hence in radius of curvature in the area of the cut, suggesting a relative homogeneity in the hoop strain across the thickness. However, previous studies with quarters of annulus dissected out [15,16] have shown annulus circumferential pre-strain dependent on radial location, with the inner annulus exhibiting large tensile strains and the outer annulus small compressive strains. It is possible that not removing the nucleus in the present study would explain the difference, where some of the hoop strain in the inner tissue was taken by the nucleus. Indeed for some of the specimens, the nucleus opened up as much as the annulus. However, removing the nucleus entirely would have required further manipulation of the tissue and reduced our confidence that no damage or over/under-hydration was caused during the delicate process of dissecting the fresh tissue. Hoop strains values determined in this work corresponds to pre-strain values reported for the inner and middle annulus in the other studies [15,16]. Moreover, with respect to the other studies, the developed method allowed for the measurement to be taken without the tissue being submitted to any freeze–thaw cycle or the intervertebral discs being stored while excised from its functional unit. Michalek *et al.* [15] reported initial openings with the nucleus fully attached to the annulus of 4.3 ± 1.8 mm, which when associated with the diameter measured in this work, corresponds to opening angles of about 20°, while the opening angles observed at 10 min relaxation in this work were 49.6° ± 9.2° with the annulus partially detached from the nucleus. The difference may come from a difference in relaxation time, from the difference between frozen and fresh tissue [30] and probably from interaction with the nucleus where it is not cut from the annulus. The measured hoop strains were not different from reported values for the end of the toe-region in tensile test of single lamellae [19]. This suggests that the extrafibrillar matrix is somewhat adapted to its mechanical state at rest. One should note that real homogeneity of residual strains throughout the radial thickness would violate static equilibrium if the tissue was a homogeneous material and it was not reported in other studies [15,16]. In the time frame of the present study, no change of local outer curvature and diameter was observed in the area of the cut. It is possible that this would have been different if the annulus had been entirely freed from the nucleus or if longer relaxation time had been allowed, measuring a pre-strain representative of a more relaxed state.

The linear assumption of the extrafibrillar matrix stress–strain behaviour in strain above hoop strain value was shown to be a reasonable hypothesis before initiation of failure. This means that a linear model for the extrafibrillar matrix of the annulus is likely to capture most of the functional behaviour. Using nonlinear extrafibrillar matrix behaviour or explicitly assigning a pre-strain to the tissue is therefore not likely to be needed in modelling the functional behaviour of the disc. However, a model of interventions including annulus surgical damage or repair would need to account for the existence of pre-strain and the non-linearity of the behaviour after an incision.

### 4.2. Finite-element models of osteodiscs

**Figure 7.**
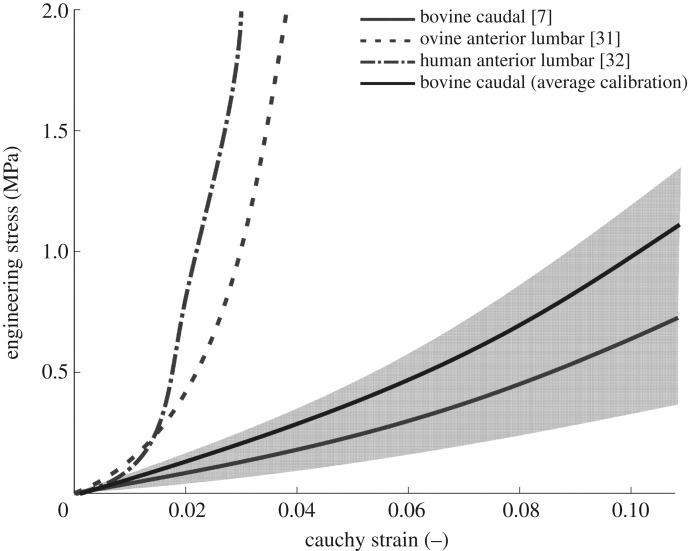
Comparison of the stress–strain behaviour obtained in the direction of the fibres for three sets of material coefficients (literature-based [7], average calibration, and specimen-specific calibration as the shaded grey area), with indication of single lamellar experimental ovine [31] and human [32] data.

Converged material parameters for each of the specimens were not too different in their values between specimens or with respect to literature values for bovine disc. However, both the specimen behaviour, measured with the RMS load difference or the stiffness values, and the material behaviour (figure 7) had largely different outcomes between specimens and with the literature [7]. Parameter values produced very different material behaviour at strains larger than 10%. Group average calibration and literature parameters led to a relatively soft behaviour at all strain values of interest whereas some of the values obtained for direct specimen calibration showed a low stiffening effect. Stiffening has been reported previously for ovine [31] and human [32] lumbar discs for individual lamellae in the fibre direction. It should be noted, however, that direct comparison of the behaviour produced in this study with individual lamellar data (figure 7) is not completely possible as our experimental data come from intervertebral disc tested *in situ*, therefore with effects of pre-strains and lamellar connections.

Using the measured functional extrafibrillar matrix behaviour and assumption about annulus compressibility and fibre orientation allowed us to use only two parameters for the annulus in the calibration and validation of the material model. Those parameters entirely describe the contribution of the annulus fibres to the behaviour of the specimens. They were calibrated using the RMS difference between computational and experimental loads, i.e. one measurement per specimen or for the group of specimens. It is possible that the optimization solution is not unique; however, the results of the validation showed improvement in the measures of interest (RMS difference and concordance of stiffness values) of similar magnitude to that of the calibration as a group. This gives confidence that the outcome of the calibration process is not only linked to the specificities of the six specimens of that group but a reflection of the whole testing and modelling process.

The material parameters calibration was performed minimizing the RMS difference on load/displacement data whereas data concordance was evaluated for the stiffness values. Even though good transition displacement and stiffness values imply good RMS difference, the opposite is not necessarily true. In this study, the initial stiffness values were not well described by the models while the transition and linear stiffness values were. This is somewhat linked to the choice of minimizing the RMS difference and not the RMS error, giving less weighting to a large relative difference at low displacement. The choice of cost function in the optimization process is therefore critical to emphasize low error for given values of interest. Moreover, the parameter *k*_2_ influences the behaviour of the model mainly in the linear region of the data, the region which has a larger weight in the relative error obtained with the model. It is therefore likely that the specific choice of cost function will give a better accuracy on *k*_2_ than it does on *k*_1_.

### 4.3. Sensitivity studies

Models were run altering the Poisson's ratio of the nucleus, in effect modifying the bulk modulus in the Mooney–Rivlin model. While a modification from full incompressibility to near incompressibility (using the bulk modulus of water) did not make any real difference in the model results, reducing the Poisson's ratio to what could be considered as almost incompressible had a very large effect in all stiffness values of the specimens. Altering the Poisson's ratio to 0.49 is in effect reducing the bulk modulus by four orders of magnitude. Modifying other parameter values in the Mooney–Rivlin model for the nucleus did not affect the specimen computational outcomes when the nucleus was considered softer; however, it slightly increased the apparent stiffness of the specimen when the nucleus was considered having a modulus of equivalent magnitude to that of the annulus. The combination of these two sensitivity studies clearly evidences the need to be able to measure the bulk modulus of the nucleus rather than its Poisson's ratio. The actual elastic modulus of the nucleus is not of major importance in models of static tests, as long as the behaviour is noticeably softer than that of the annulus. The models showed some sensitivity to the reconstructed geometry of the intervertebral disc, the variation in the mechanical behaviour due to anatomical variation was much lower than the variation between specimens. A non-user-dependent protocol to create intervertebral disc geometry from CT images where the soft tissues are not discernible is therefore a good approximation of the actual effects of the geometry. The nucleus-to-annulus size ratio is of critical importance to predict the transition and linear stiffness values of the models, i.e. at medium to high strains, and for a large nucleus, where both the sensitivity and the discrepancy with standard ratio are higher than for the initial stiffness at lower strain values. Given that no clear anatomical boundaries can be observed between nucleus and annulus, modelling a smooth transition between annulus and nucleus, e.g. including differentiation between inner and outer annulus [33], may be the best approach.

### 4.4. Direct-controlled calibration and validation

In this study, we discussed the effect of direct-controlled calibration and validation of finite-element models, i.e. specimen-specific models for which geometry and experimental data are known and directly compared (direct) and acquired in conditions that can be exactly replicated (controlled). Only two parameters were calibrated, using data from six specimens. The calibration and validation processes had the exact same experimental and computational methodologies on two sets of independent specimens.

A similar study [34] using ovine tissue calibrated and validated an annulus model using a GOH material. They performed measurement of the extrafibrillar matrix compressive behaviour, as well as performing controlled calibration of one finite-element model of 20 dissected portions of the annulus tested in axial tension. Given the calibration of the annulus in tension, the nonlinear behaviour of the fibres is more prominent to the specimen behaviour, resulting in a ratio *k*_1_/*k*_2_ lower than those reported here. Other studies that performed direct-controlled calibration of intervertebral disc behaviour for more than one specimen either assumed axial symmetry of bovine discs and relatively low axial compressive strains [8] or used a relatively low numbers of human discs (three) for a large number of unknowns (six parameters, varying with the degree of degeneration of each disc) [4]. For bovine discs, the axial symmetry is a relatively weak assumption, with the endplates showing different concavity in the proximal and distal sides. Moreover, at larger strain levels, several of the assumptions in Newell *et al*. [8], such as linearity of the fibres or rigidity of the bone, are no longer valid. Previous work validating finite-element models of functional spinal units had shown that nonlinear material models were required at axial compression higher than 15% apparent strains [5].

While both Malandrino *et al*. [4] and Casaroli *et al*. [34] performed direct calibration of their models, their validation process was looking at an entirely different model type (three L3-L4 discs in calibration versus one L1-S1 segment for validation and 20 lumbar annulus portions versus one L3-L4 functional unit respectively), and measured different outcomes than in the calibration process (global creep response versus segmental ranges of motions and tensile behaviour versus otherwise published range of motion respectively). Such an approach to validation provides some confidence that the calibrated parameters can be used in other models but does not demonstrate the reliability of those parameters to replicate in other specimens the tests for which they have been calibrated.

The present *in silico* models and *in vitro* tests were quasi-static tests in axial compression. They were not meant to represent *in vivo* conditions or mechanical behaviour under any other loading conditions, specifically under flexion-extension where the effect of the fibre non-linearity will be more significant. The work provides a framework to calibrate material parameters that can be extended to more realistic models, in particular for intervertebral disc, inclusion of viscous effects and control of hydration are of large importance for most applications.

## 5. Conclusion

This work measured hoop strains in fresh bovine intervertebral discs and showed that the behaviour of the extrafibrillar matrix of the annulus is relatively linear within the physiological strain range. In computational models that aim to represent the healthy functional behaviour of the disc, it is unlikely that including the non-linearity of the extrafibrillar matrix is needed.

The sensitivity study performed on the nucleus assumptions showed that, for static compression, attention is needed to define the compressibility accurately, rather than the Poisson's ratio where a difference between 0.49 and 0.4999 would be difficult to measure. Moreover, when large strain behaviour is of interest, accounting for the difference in behaviour between inner and outer annulus may be necessary to better capture the anatomical and material transition between annulus and nucleus.

Performing direct-controlled calibration and validation of material parameters accounts for the whole process of experimental design and *in silico* model methodology. The outcome reflects the state of the tissue (pre-strain or stress, microstructure, level of hydration), the testing conditions, the geometry acquisition, and the model development methodology. Direct-controlled calibration and validation for computational models of natural tissue is a process that provides confidence that the model development methodology can capture the measurable variation within the population and not only its average behaviour. It is a key step for stratification of interventions or disease progression prediction through *in silico* modelling.

## Data accessibility

All new raw and processed data produced in this study are available openly from the University of Leeds data repository [27]. The reverse finite-element tools used in this work are accessible at https://doi.org/10.5281/zenodo.557057 [25] and https://doi.org/10.5281/zenodo.20601. Statistical scripts are available with the main data package and have been processed with the R Project for Statistical Computing (https://www.r-project.org/).
